# Size Effects of the Anions in the Ionothermal Synthesis of Carbon Nitride Materials

**DOI:** 10.1002/chem.202200705

**Published:** 2022-05-02

**Authors:** David Burmeister, Johannes Müller, Julian Plaickner, Zdravko Kochovski, Emil J. W. List‐Kratochvil, Michael J. Bojdys

**Affiliations:** ^1^ Department of Chemistry & IRIS Adlershof Humboldt-Universität zu Berlin Brook-Taylor-Str. 6 12489 Berlin Germany; ^2^ Department of Chemistry King's College London Britannia House Guy's Campus 7 Trinity Street London SE1 1DB UK; ^3^ Department of Physics & IRIS Adlershof Humboldt-Universität zu Berlin Newtonstraße 15 12489 Berlin Germany; ^4^ Helmholtz-Zentrum Berlin für Materialien und Energie GmbH Hahn-Meitner-Platz 1 14109 Berlin Germany; ^5^ Leibniz-Institut für Analytische Wissenschaften – IAS e.V. Schwarzschildstrasse 8 12489 Berlin Germany; ^6^ Institute of Electrochemical Energy Storage Helmholtz-Zentrum Berlin für Materialien und Energie Hahn-Meitner-Platz 1 14109 Berlin Germany

**Keywords:** covalent organic frameworks, graphitic carbon nitride, ionothermal synthesis, layered materials, metal free semiconductors

## Abstract

Semiconducting carbon nitride polymers are used in metal‐free photocatalysts and in opto‐electronic devices. Conventionally, they are obtained using thermal and ionothermal syntheses in inscrutable, closed systems and therefore, their condensation behavior is poorly understood. Here, the synthetic protocols and properties are compared for two types of carbon nitride materials – 2D layered poly(triazine imide) (PTI) and hydrogen‐bonded melem hydrate – obtained from three low‐melting salt eutectics taken from the systematic series of the alkali metal halides: LiCl/KCl, LiBr/KBr, and LiI/KI. The size of the anion plays a significant role in the formation process of the condensed carbon nitride polymers, and it suggests a strong templating effect. The smaller anions (chloride and bromide) become incorporated into triazine (C_3_N_3_)‐based PTI frameworks. The larger iodide does not stabilize the formation of a triazine‐based polymer, but instead it leads to the formation of the heptazine (C_6_N_7_)‐based hydrogen‐bonded melem hydrate as the main crystalline phase. Melem hydrate, obtained as single‐crystalline powders, was compared with PTI in photocatalytic hydrogen evolution from water and in an OLED device. Further, the emergence of each carbon nitride species from its corresponding salt eutectic was rationalized via density functional theory calculations. This study highlights the possibilities to further tailor the properties of eutectic salt melts for ionothermal synthesis of organic functional materials.

## Graphical abstract

**Size effects**: Ionothermal condensation of dicyandiamide in alkali halide salt melts leads to the formation of extended 2D, layered frameworks only in the presence of small halides such as chloride, and bromide. With increasing size of the alkali halide intercalate, stabilizing van der Waals interactions between extended, π‐conjugated triazine‐based sheets are lost. The main, crystalline product from an alkali iodide eutectic was identified as melem hydrate, a heptazine (C_6_N_7_)‐based, hydrogen‐bonded, monoclinic solid.

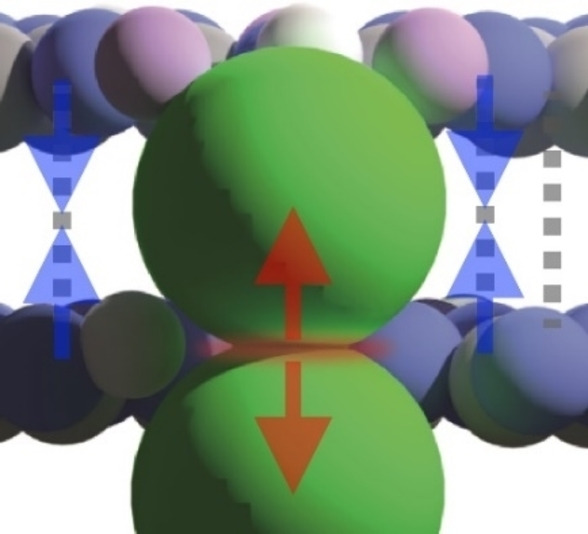

## Introduction

**Figure 1:**
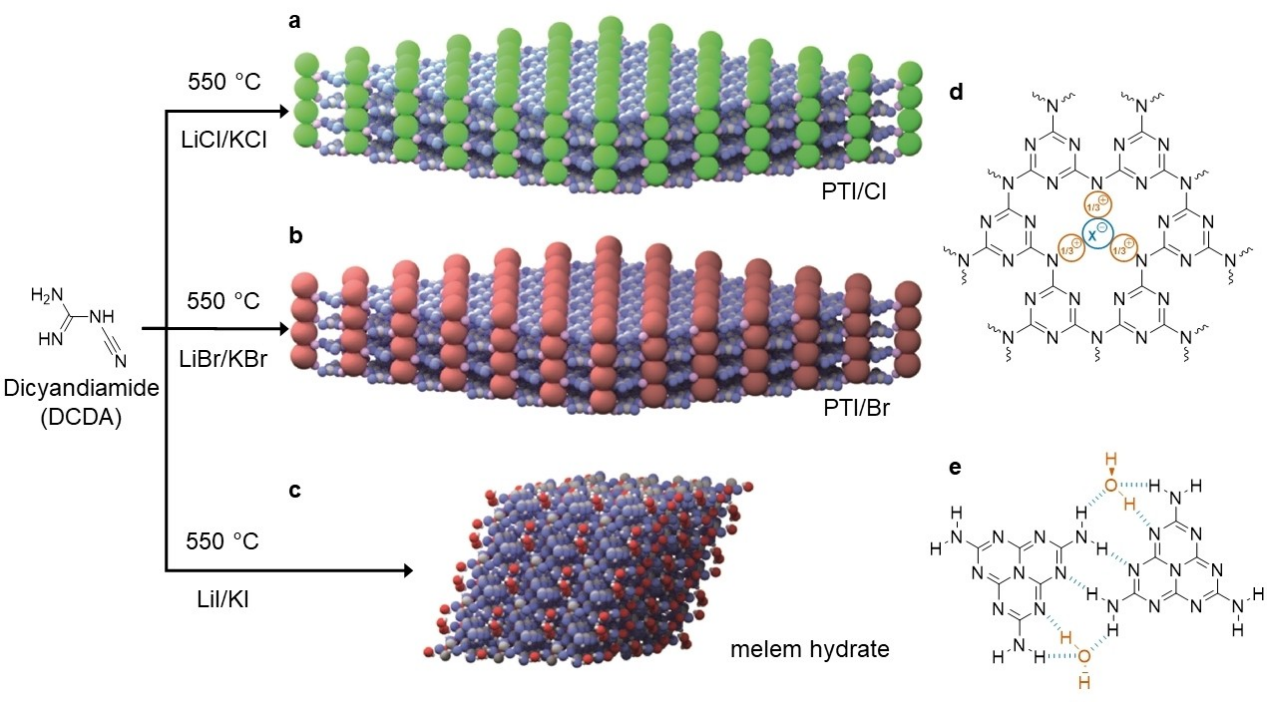
Condensation products of dicyandiamide (DCDA) in different eutectic salt melts: a) poly(triazine imide) with intercalated chloride (PTI/Cl), b) poly(triazine imide) with intercalated bromide (PTI/Br), and c) melem hydrate (C_6_H_6_N_10_ ⋅ H_2_O). d) The poly(triazine imide) network forms triangular cavities with inclusion of halides (X^−^, in blue) counterbalanced by lithium (in orange). e) Melem hydrate forms an extended hydrogen‐bonded network (in blue) between melem (C_6_H_6_N_10_) units and water (in orange) in three‐dimensions.

Carbon nitride materials have emerged as interesting and chemically tunable,[^1^, ^2^, ^3^, ^4^, ^5^, ^6^, ^7^, ^8^, ^9^, ^10^] metal‐free semiconductors with applications in heterogenous photocatalysis,[^11^, ^12^, ^13^, ^14^, ^15^] and most recently as active materials in organic light‐emitting diodes.[^16^, ^17^, ^18^] Conventional synthetic protocols for carbon nitride materials center on high‐pressure decomposition of CNH‐containing molecular,[^19^, ^20^] ion and vapor deposition of nitrogen ions and carbon,^[21]^ plasma decomposition of methane and N_2_,^[22]^ shock wave compression,^[23]^ sputtering experiments, pulsed laser ablation of graphite in combination with an atomic nitrogen source, and electrochemical approaches.[^24^, ^25^] Diffusion‐controlled methods do not yield high quality products, as the formation of the crystalline product is impeded by atom displacement and knock‐on collisions.[^6^, ^26^] In contrast, poly(triazine imide) – or PTI for short – is the most well‐defined, crystalline form of graphitic carbon nitrides, and it is obtained via ionothermal condensation of dicyandiamide (DCDA, C_2_H_4_N_4_) in eutectic salt melts.[^1^, ^26^] PTI is a 2D material with layers built from covalently‐linked triazine (C_3_N_3_)‐units that form triangular pores into which anions (chloride and bromide) and lithium cations from the salt melt become intercalated selectively (Figure 1a, b, d).^[26]^ The snug fit of the halide anions into the pores of layered PTI suggests a strong templating effect. Eutectic salt melts control the structure of the carbon nitride materials formed in ionothermal synthesis as they act as polar solvents with extremely high boiling points for organic mono‐ and oligomers.[^1^, ^26^] To‐date, however, the role of the anions in the ionothermal formation of carbon nitrides has not been elucidated.

In this study, we investigate systematically the influence of three eutectic salt melts with increasing anion sizes (Cl^−^<Br^−^ < I^−^) on the formation of carbon nitride materials during ionothermal synthesis, namely: (i) LiCl/KCl (45 wt%/55 wt%, m.p. 352 °C), (ii) LiBr/KBr (52 wt%/48 wt%, m.p. 348 °C), and (iii) LiI/KI (58 wt/42 wt%, m.p. 285 °C).^[27]^

We consider the known chloride‐ and bromide‐intercalates of poly(triazine imide) and compare them with the hitherto unknown product from the iodide eutectic. The carbon nitride material from the LiI/KI salt melt is obtained as a powder from which we isolate single crystallites for structural analysis using single crystal X‐ray diffraction (sc‐XRD). The isolated single crystals were identified as melem hydrate (Figure 1c, e). Using the iodide eutectic as the medium for ionothermal synthesis, we did not observe the formation of a crystalline extended polymer with a higher degree of condensation. This finding strongly indicates that the smaller anions (Cl^−^ and Br^−^) are stabilizing the formation of poly(triazine imide) layers and facilitate their π‐π stacking, as confirmed by density functional theory calculations (CASTEP).

## Results and Discussion

### Experimental design

**Figure 2:**
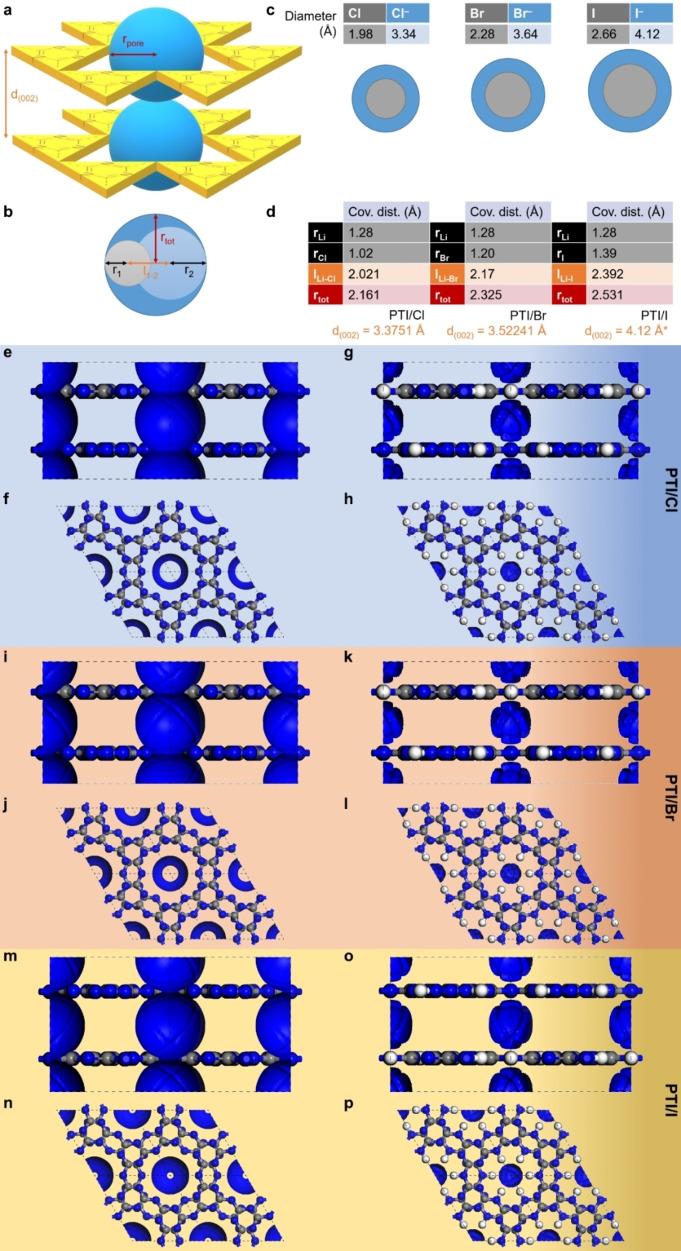
Considerations of available free volume for intercalation of lithium halide species in poly(triazine imide) frameworks. a) Principally available space in a PTI‐based framework is confined by the pore diameter, r_pore_, and the galley height, d_(002)_. b) Representation of the dynamic covalent radius, r_tot_, of a lithium halide. c) Diameters of halogen atoms and their respective halides. d) Table above, calculation of the dynamic covalent radii of lithium chloride, lithium bromide, and lithium iodide. Below, gallery heights for poly(triazine imide) model structures with intercalated lithium halides, derived from experiment for PTI/Cl and PTI/Br and, and estimated for PTI/I (denoted by *) on the basis of the iodide radius. Connolly surface areas (in blue) generated for PTI frameworks with removed hydrogens (e, f) and without (g, h) using the dynamic covalent radius of LiCl as probe molecule. In analogy, Connolly surface areas generated for PTI frameworks using LiBr (i‐l) and LiI (m‐p) as probe molecules, respectively.

The poly(triazine imide) (PTI) framework provides two types of potentially accessible volumes for lithium halide intercalates: (i) within the pores defined by the in‐plane, covalent bonding pattern of covalently linked triazine (C_3_N_3_)‐units (Figure 2, a; blue spheres with radius, r_pore_), and (ii) in the galleries between π‐π stacked PTI layers (Figure 2, a; with distance, d_(002)_). Since the material is overall charge‐neutral, we assume intercalation of a lithium halide species with a total, dynamic radius, r_tot_, that may not exceed the pore diameter, r_pore_ (Figure 2, b). The gallery height for stacked PTI sheets is known to be 3.3751 Å for PTI/Cl and 3.52241 Å for PTI/Br,[^1^, ^26^] which correlates with the ionic diameters for chloride (3.34 Å) and for bromide (3.64 Å). Hence, for a hypothetical PTI/I we assume that the gallery height does not exceed d_(002)_=4.12 Å which is the radius of iodide (Figure 2, c). Using standard values from the Computational Chemistry Comparison and Benchmark Data Base (CCCBDB), we obtain dynamic radii of lithium halides as follows: 2.161 Å for LiCl, 2.325 Å for LiBr, and 2.531 Å for LiI (Figure 2, d). These will serve as probe radii for the generation of Connolly surfaces. We further consider the two extreme cases in which (i) the bridging NH‐groups are fully deprotonated and become available for isoelectronic replacement by lithium cations (Figure 2, e, f, i, j, m, n) and (ii) the bridging NH‐groups remain fully protonated (Figure 2, g, h, k, l, o, p). Examining the Connolly surfaces generated using these constraints (Figure 2, e‐p), we observe that lithium halides can in principle be intercalated into the interstitial spaces between the sheets above and below the pores of all PTI structures. In the case that bridging NH‐groups are isoelectronically converted to NLi‐groups, sufficiently large free volumes exist in the pores of PTI/Cl and PTI/Br to allow in‐plane incorporation. However, LiI is too large. In the case that bridging NH‐groups remain unchanged, there appears to be no free volume within the pores, but all lithium halides could potentially be situated in the interstitial spaces above and below.

The expected layer‐stacking distance for PTI/I of≥4.12 Å that is needed to accommodate a pillared arrangement of iodide species is exceptionally large for an aromatic, discotic system (cf. graphite 3.35 Å, PTI/Cl 3.38 Å, PTI/Br 3.52 Å).^[1]^ Hence, as a working‐hypothesis, we assume that a poly(triazine imide) with intercalated LiI will not form and that, instead, a synthesis in LiI/KI will conceivably lead to (i) an intercalate‐free PTI, (ii) a PTI with a stacking mode other than pore‐on‐pore, or (iii) a different material entirely.

### Characterization and identification of the crystalline product from the iodide eutectic

**Figure 3:**
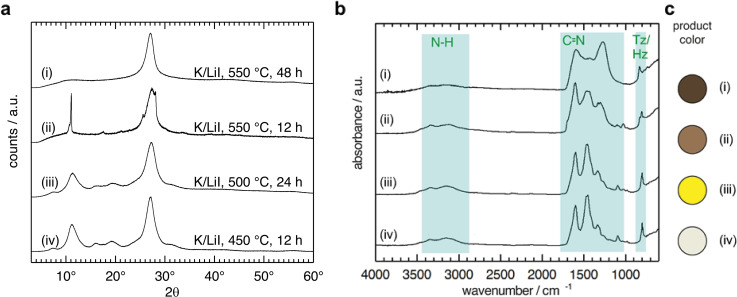
Powder X‐ray diffraction (PXRD) data (a), Fourier transform‐infrared (FTIR) spectra (b), and product colors (c) of carbon nitride samples obtained at various conditions in a LiI/KI eutectic (i‐iv).

The ionothermal condensation of DCDA in LiI/KI eutectic was set‐up according to literature, in analogy to existing protocols for PTI/Cl and PTI/Br,[^16^, ^28^] and as outlined in the Supporting Information. We screened reaction conditions with different temperatures between 450 °C and 550 °C and with reaction times from 12 h to 48 h. We present an overview of the powder X‐ray diffraction (PXRD) and Fourier transform‐infrared spectroscopy (FTIR) data for all additional products obtained at various reaction conditions in the Supporting Information section (Figure S1). Products obtained at temperatures below 550 °C show broad peaks in the diffractogram (Figure 3, a), indicating that the condensation process is still incomplete. The color of the powders obtained in the range from 450–500 °C changes from white to bright yellow as temperature is increasing. FTIR spectra show the presence of NH_2_ at 3460 and 3350 cm^−1^ signals and a peak at 796 cm^−1^ attributed to the out‐of‐plane breathing‐mode of triazines (C_3_N_3_, Tz) and heptazines (C_6_N_7_, Hz) (Figure 3, b).^[29]^

The elemental composition of products obtained at temperatures up to 500 °C for 24 h fits with the CN ratio of melem (Table 1, Table S1). Extended reaction times (e. g. 48 h) and higher temperatures (e. g. 550 °C) yield samples with drastically increased carbon content, leading to a darker coloration and an amorphization of the product (Figure 3, a (i)). Compared to crystalline PTI/Br that is obtained as a beige powder after 48 h at 550 °C,^[1]^ the early onset of carbonization in LiI/KI eutectics indicates that the formation of a more temperature stable, crystalline poly(triazine imide) structure is disfavored due to a lack of stabilizing anions of appropriate size. Moreover, instead of the intercalation of alkali iodide and the appearance of a PTI‐type diffraction pattern (Figure 3, a), we observe the formation of iodine vapors inside the quartz ampoule at temperatures above 480 °C. This indicates that in the iodide eutectic DCDA reaction mix unidentified redox processes are ongoing at these temperatures (Figure S4). In comparison, using bromine and chlorine eutectics at temperatures up to 600 °C, we observed no formation of halogen gases.

**Table 1:** Combustion elemental analysis of products obtained from the LiI/KI eutectic at various reaction conditions (i‐iv). The CN ratio of the products is compared to the expected CN ratio of melem.

Reaction conditions	C : N
(i) LiI/KI, 550 °C, 48 h	0.55:0.45
(ii) LiI/KI, 550 °C, 12 h	0.46:0.54
(iii) LiI/KI, 500 °C, 24 h	0.38:0.62
(iv) LiI/KI, 450 °C, 12 h	0.38:0.62
melem (theoretical)	0.38:0.62

^13^C cross‐polarization magic‐angle‐spinning (CP/MAS) solid‐state NMR spectra of the products show bands at 154 ppm and 164 ppm in good agreement with the reported values for melem at 155 ppm and 164 ppm (Figure S2).^[29]^ ^15^N solid‐state NMR has two main features at 204 ppm and 280 ppm that we assign to the pyridinic nitrogen and the amine nitrogen environments reported for melem (at 205 ppm and 281 ppm).^[29]^ A weak band at 259 ppm is assigned to amorphous condensation products containing secondary amines that is structurally similar to short chains of condensed melem units (so‐called “melon”). When compared to melon, the NH_2_ (265 ppm) and NH (245 ppm) nitrogen bands are shifted towards higher ppm values.^[30]^ The photoluminescence (PL) spectrum of the product obtained at 450 °C has a maximum at 385 nm which coincides with the PL data obtained for pure melem (Figure S3.).^[29]^ With increasing temperature, the PL maximum shifts towards higher wavelengths. We interpret this shift as an increasing contribution of the amorphous, brown phase that we believe to be disordered, partially carbonized melon‐like structures.[^30^, ^31^] In summary, samples obtained at temperatures up to 500 °C contain melem as the main phase product. At a temperature of approx. 550 °C, we observe the onset of carbonization of the organic components.

**Figure 4:**
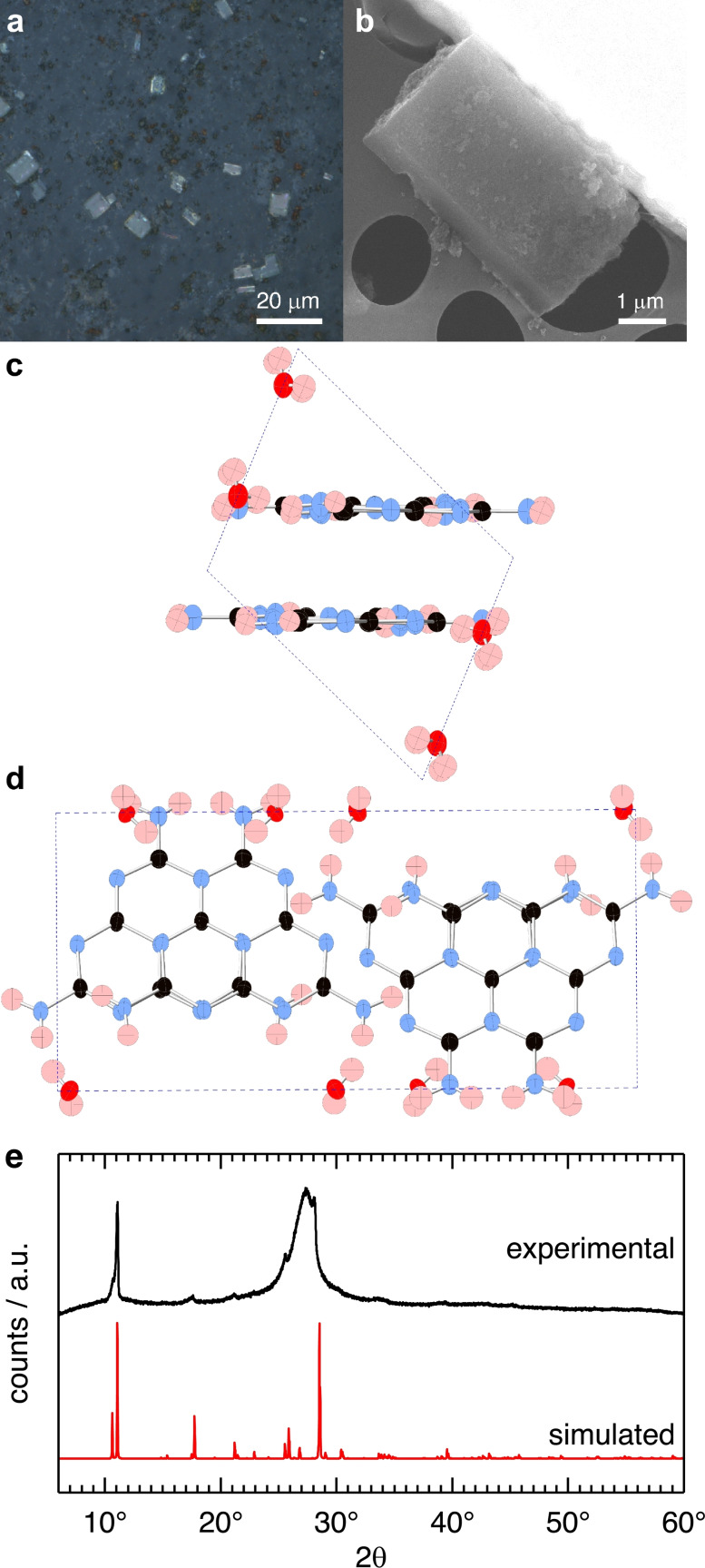
Structural characterization of melem hydrate crystals obtained at 550 °C and 12 h in the LiI/KI eutectic salt melt. a) Light microscopy image of the bulk product on scotch tape shows two morphologies distinguishable as brown, amorphous particles and rectangular crystallites. b) Scanning electron microscopy (SEM) (20 kV accelerating voltage) image of a single crystallite. Ball‐and‐stick representation of the unit‐cell of melem hydrate obtained from single‐crystal X‐ray diffraction projected along c) the b‐axis and d) the c‐axis, respectively. Atomic positions are marked as thermal ellipsoids, colored red for oxygen, blue for nitrogen, black for carbon and light red for hydrogen. Hydrogen positions are approximated. e) Comparison of the powder X‐ray diffraction (PXRD) pattern of the bulk product (in black) and the simulated diffraction pattern of melem hydrate based on the structural data from single crystal X‐ray diffraction data (in red).

We selected the carbon nitride material with the highest, apparent crystallinity (LiI/KI, 550 °C, 12 h; Figure 3, (ii)) for detailed investigations. Light microscopy reveals two phases: a brown phase with no geometric order and a transparent phase with rectangular crystallites (Figure 4, a). The crystals were imaged by scanning electron microscopy (SEM) (Figure 4, b) showing characteristic, sharp edges, and a rectangular shape.

Energy dispersive X‐ray spectroscopy (EDX) of a crystallite corroborates the results of the combustion elemental analysis: the main elements are nitrogen (65 at%) and carbon (35 at%). Contributions from salt residues and carbonization are evident only in the amorphous, brown phase of the product (Figure S5). Notably, we observe no evidence of iodide intercalation in the carbon nitride crystals. To prove that the sample is also free of alkali metals, we performed inductively coupled plasma‐optical emission spectroscopy (ICP‐OES) and X‐ray photoelectron spectroscopy (XPS). The condensation product obtained at 550 °C, 12 h contains 0.38 wt% Lithium and 0.73 wt% potassium. These residual amounts of alkali metals are low and non‐stoichiometric. They are most likely inclusions situated in the amorphous, brown phase and cannot be washed out by hot water. XPS results confirm the CN‐ratios observed in combustion elemental analysis and in EDX and show no salt contributions (Figure S6). UV‐Raman spectra show indicative bands for heptazines at 1661 cm^−1^ (w), 1571 cm^−1^ (w), 1155 cm^−1^ (s) and 545 cm^−1^ (s) in the translucent crystals (Figure S7).^[29]^ Strong bands at 980 cm^−1^ (s), 1401 cm^−1^ (s) and 1310 cm^−1^ (s) are unassigned. Raman bands in the amorphous, brown phase are weaker and broader compared to the spectra obtained for the crystals, as is anticipated for a highly disordered phase.

We were able to solve the crystal structure of the crystalline phase using single crystal X‐ray diffraction. In this experiment, we used a micro actuator to pick up and transfer the micro‐sized crystallites (Figure S8). The structure solution revealed the crystallites to be melem hydrate (C_6_N_7_(NH_2_)_3_ ⋅ H_2_O) (Table S2). The stacking distance of the melem units is 3.2 Å at slight offset to maximize dipole‐dipole interactions (Figure 4, c). Melem hydrate crystals are held together by a three‐dimensional network of hydrogen bonds with lengths of ∼2.2 Å between (i) pyridinic nitrogen atoms of the heptazine cores and adjacent amine groups and (ii) water molecules and amine groups (Figure 4, d). In contrast, melem crystals obtained via sublimation are not aligned in co‐planar layers.^[29]^ The refined structure from the single crystal experiment is in good agreement with the PXRD profile of the bulk material (Figure 4, e). We attribute the additional broad peak at 27° 2θ to the emergence of the glassy, secondary brown phase that is formed because of carbonization of crystalline melem hydrate into randomly stacked, π‐conjugated fragments. A similar behavior was reported previously for carbonization products of poly(triazine imide) in a LiBr/KBr eutectic.^[16]^ As expected, melem hydrate undergoes a reversible phase change to melem during vacuum drying at 200 °C for 24 h (Figure S9).^[29]^

Scanning force microscopy (SFM) shows a high degree of order at the surface of melem hydrate single crystals (Figure S10). The observed periodicity is in good agreement with the Connolly surface of a melem hydrate crystal cleaved at the 001 plane. High resolution cryo‐transmission electron microscopy (HR‐TEM) images show periodic lattice fringes with 0.8 nm distances that correspond to the 001 crystallographic plane of melem hydrate (Figure S11).

In analogy to PTI‐based organic light‐emitting diodes (OLEDs),^[16]^ we have constructed a single layer organic light emitting device from the product obtained at 500 °C, 12 h in LiI/KI. The device shows a steep diode like onset at 4.5 V in forward bias direction. At 13 V we observed a maximum luminance of only 1 cd m^−2^ (Figure S12). The Brunauer‐Emmett‐Teller (BET) surface area was determined by nitrogen adsorption‐desorption isotherms of the 450 °C and 550 °C products showing no micropores and a low surface area of 57 and 71 m^2^ g^−1^ respectively (Figure S13). In addition, we tested the hydrogen evolution reaction (HER) activity of products from 500 °C, 12 h and 550 °C, 12 h with triethanolamine as sacrificial agent under xenon lamp illumination without cutoff filter and PtCl_6_ as co catalyst. Unlike PTI and polymeric carbon nitride,[^25^, ^32^, ^33^, ^34^] we did not observe any detectable HER activity for dispersed melem hydrate.

### Thermodynamic considerations of product stability in different salt melts

**Figure 5:**
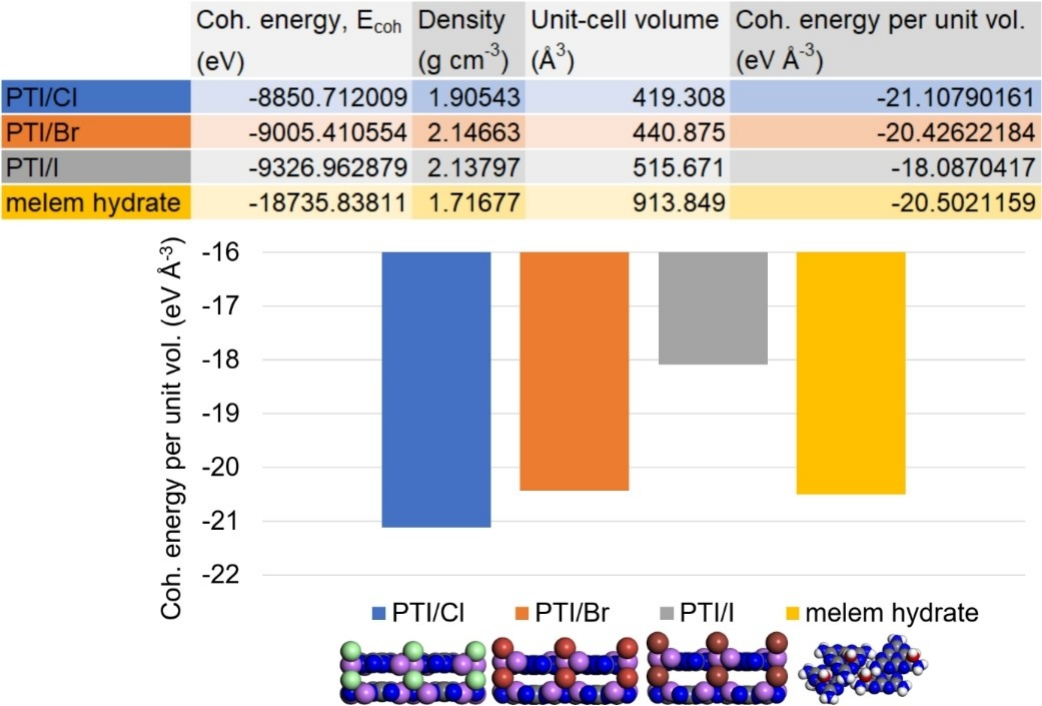
Results of ab initio total energy density functional theory (DFT) calculations using a plane wave basis set (CASTEP; energy cut‐off 450 eV; exchange correlation PBE‐GGA; atomic relaxation until force/atom <0.02 eV Å^−1^) for the four periodic carbon nitride materials PTI/Cl (in blue), PTI/Br (in orange), PTI/I (in grey), and melem hydrate (in gold).

In contrast to the poly(triazine imide) intercalates obtained in LiCl/KCl and LiBr/KBr, we see that the ionothermal condensation of dicyandiamide in LiI/KI leads to a thermally less‐stable melem hydrate as the main, crystalline product – and this although the interstitial cavities of the layered PTI framework would be sufficiently large to accommodate LiI in principle (Figure 2). Analogous reactions in an open crucible set‐up using DCDA and a LiI/KI eutectic similarly found that no poly(triazine imide) structures are formed.^[35]^ Our work and previous reports indicate that the condensation reaction is halted at melem, the precursor to the formation of poly(triazine imide) – irrespective of whether an open or closed reaction set‐up is chosen.^[36]^ We performed ab initio total energy density functional theory (DFT) calculations to compare the stabilities of the hitherto known main crystalline products from ionothermal, salt‐mediated syntheses – PTI/Cl and PTI/Br – with the stabilities of a hypothetical PTI/I crystal and the identified melem hydrate phase (CASTEP; plane wave basis set; energy cut‐off 450 eV; exchange correlation PBE‐GGA; atomic relaxation until force/atom <0.02 eV Å^−1^) (Figure 5).^[37]^ Due to the differences in unit‐cell volumes and content, we have normalized the obtained cohesive energies, E_coh_, per unit volume and used these values as a guide for material stability. We find that in the logical series of lithium halides LiCl → LiBr → LiI, the corresponding poly(triazine imide) intercalation compounds become less stable following a trend in stability of: PTI/Cl>PTI/Br>PTI/I. In the LiI/KI eutectic, melem hydrate is more stable than a hypothetical PTI/I framework, as observed in the experiment.

Based on our initial hypothesis, we pointed out the infeasibly large layer‐stacking distance between PTI‐sheets of≥4.12 Å that is needed to accommodate a pillared arrangement of lithium iodide species. Our DFT calculations support that the artificially imposed, large gallery‐height in a PTI/I structure chiefly contributes to a loss in cohesive van der Waals interactions between π‐aromatic layers and disfavors the formation of a poly(triazine imide) framework. This calculation does not consider conceivable, energetic contributions from ionic stabilization of triazine‐based intermediates along the reaction pathways, but it assists in rationalizing the observed selectivity of experimentally observed products.

## Conclusion

In summary, we identified the main, crystalline product obtained from the ionothermal condensation of dicyandiamide in a LiI/KI eutectic salt melt as melem hydrate (C_6_N_7_(NH_2_)_3_ ⋅ H_2_O). This stands in contrast to poly(triazine imide) frameworks with intercalated lithium halides obtained from LiCl/KCl and LiBr/KBr eutectics. Glassy melem is the main product at temperatures below 500 °C, and transparent, monoclinic melem crystals are obtained at 550 °C (P2_1_/c, a=8.6040(17) Å, b=16.630(3) Å, c=6.8840(14) Å, β=111.91(3)). In contrast to extended carbon nitride polymers, an OLED with melem powders as the active material shows only low luminance of 1 cd m^−2^, and melem hydrate has no detectable activity as a photocatalyst in the hydrogen evolution reaction.

Unlike in LiCl/KCl and LiBr/KBr eutectics, we observe no selective, stoichiometric intercalation of lithium iodide into a 2D, extended triazine‐based framework in the final product. DFT calculations corroborate that the pillared intercalation of lithium halides leads to a loss of stabilizing van der Waals interactions between extended, π‐conjugated triazine‐based sheets as the size of the intercalate increases (LiCl < LiBr < LiI), and ultimately to the selection of a thermodynamically more favourable, monomeric, heptazine‐based product.

This study highlights the importance of anion‐size effects in the ionothermal synthesis of π‐conjugated organic materials, and it showcases a computationally‐assisted methodology for future experiments that (i) use novel, binary and ternary eutectic salt melts, and (ii) aim for thermodynamic selectivity of reaction pathways and products.

## Supporting Information

Supporting Information is available from the Wiley Online Library or from the author.

Deposition Number 2095271 contains the supplementary crystallographic data for this paper. These data are provided free of charge by the joint Cambridge Crystallographic Data Centre and Fachinformationszentrum Karlsruhe Access Structures service.

## Conflict of interest

The authors declare no conflict of interest.

1

## Supporting information

As a service to our authors and readers, this journal provides supporting information supplied by the authors. Such materials are peer reviewed and may be re‐organized for online delivery, but are not copy‐edited or typeset. Technical support issues arising from supporting information (other than missing files) should be addressed to the authors.

Supporting Information

## Data Availability

The data that support the findings of this study are openly available in Zenodo at https://doi.org/10.5281/zenodo.6327947, reference number 6327947.
