# Supplementary material for: Size Effects of the Anions in the Ionothermal Synthesis of Carbon Nitride Materials
Source: Chemistry. 2022 May 2;28(33):e202200705. doi: 10.1002/chem.202200705 (PMC9321126; doi:10.1002/chem.202200705)
Supplement: Supplementary file 1 — Supporting Information [file CHEM-28-0-s001.pdf]

Chemistry–A European Journal

Supporting Information

Size Effects of the Anions in the Ionothermal Synthesis of Carbon Nitride Materials

David Burmeister, Johannes Müller, Julian Plaickner, Zdravko Kochovski, Emil J. W. List-Kratochvil, and Michael J. Bojdys*

Experimental

Experiments **have to be conducted with caution** since they employ glassware under high pressure and temperature. Furthermore formation of **explosive compounds** like NI_3 can occur.

Dicyandiamide condensation in iodide eutectic salt melts: 1 g of precursor dicyandiamide (DCDA, Sigma Aldrich >99%) is ground together with an eutectic salt mixture of LiI and KI (lithium iodide, anhydrous Sigma Aldrich >99.99%, potassium iodide, anhydrous Sigma Aldrich >99%) (15 g; 58:42 wt%, m.p. 285 °C) in a glovebox. The reaction mixture is filled into a quartz ampule and sealed. The sealed quartz ampule is placed into a furnace (Nabertherm, L 5/11/B180, 2.4 kW) at 400 °C for 4 h. Then the temperature is increased (10 K/min) to 480 °C and held for 2 h. Then the temperature in increased to the final reaction temperature for the time frames stated in the document. At around 450 °C noticeable amounts of Iodine are in the gasphase in the ampule. The furnace is then allowed to cool naturally. The ampule is removed at room temperature, opened, and the salt block is dissolved in Milli-Q water in a 50 mL Falcon. The slurry is centrifuged and the supernatant is decanted. The pellet is resuspended in hot water on a shaker and centrifuged again. The supernatant is decanted and the process is repeated for two times with hot water and two times with methanol (>99% for synthesis). The resulting pellet is resuspended in methanol, transferred into vacuum safe glas ware and the methanol is evaporated. The resulting powder is dried under vacuum at 200 °C for 24 h before combustion analysis. Assuming the product of the synthesis at 450 °C is melem, the yield is 58% (0.5 g).

Preparation of suspensions for TEM, SEM, XPS, Raman sample preparation: For the dispersion 10-20 mg of product are redispersed in 5mL of EtOH. The dispersion is then dropcasted either on a TEM grid (Plano, S147-2, holey carbon) or a wafer for TEM/SEM (1 µL) or a 1x1 cm indium tin oxide coated glas substrate for XPS (10-50 µL).

Sonication of CN suspensions for preparation of OLEDs: Sonication of CN powder was conducted with a BANDELIN HD 2200/-U (200 W, HF 20 kHz) sonotrode setup with a MS72 sonotrode with the amplitude set to 10% in continuous mode. The falcon tube is placed in an ice bath and the sonotrode was immersed about 3-5 mm into the suspension. Suspensions are sonicated for 3 h, centrifuged at 7690 g for 1 min to reduce large particles and the supernatant was removed with a pipet into a falcon.

OLED preparation: ITO-coated glass substrates (sheet resistance = 20 Ω per square) were cleaned by sequential sonication (10 minutes) in (i) acetone and (ii) isopropanol followed by drying *via* a nitrogen gun. The substrates were then treated *via* O_2 plasma (partial pressure 1.2 × 10^{-1} mbar) for 15 minutes at 10.2 W. 50 nm PEDOT:PSS (Osilla) films were spin coated as hole injection layer and heated to 220 °C for 10 min. 40 µL CN dispersion was drop cast at 60 C from the chlorobenzene dispersion onto the pixel areas of the PEDOT:PSS covered substrate (ca. 150 mm^2). 5 nm Calcium and 200 nm Aluminium were evaporated in a PVD chamber at 10^{-5} mbar. Finally the OLEDs were encapsulated with UV-curable resin (Osilla) and a glass slide. Current density–voltage–luminance characterization was performed with a Keithley 2612B source meter and a Konica Minolta LS-160 luminance meter in a purpose-built setup. Electroluminescence spectra were taken with a CS2000 spectrometer (Ocean Optics) using OceanView software.

Fourier transform infrared (FT-IR): Spectra were recorded from solid on a Thermo Scientific Nicolet iS5 spectrometer (Thermo Fisher Scientific, Waltham, MA, USA) in the wavenumber range of 4000-600 cm^{-1} with resolution of 4 cm^{-1}.

Raman: UV Raman spectra were recorded with a Horiba T64000 spectrometer in single-grating mode. The excitation source was provided by a diode-pumped solid-state laser from CryLas at a wavelength of 266 nm and power of 4 mW. The light was focused on the sample

with a Thorlabs LMU-40x-UVB objective (backscattering geometry). A notch filter with cut-off at 220 cm^{-1} was used to filter out the elastically scattered light. The acquired Raman spectra were calibrated by comparison with the spectrum of a Ga_2O_3 crystal by using a quadratic calibration curve. This procedure allows for reduction of the experimental error at approximately 5 cm^{-1}, which is below the spectral resolution of 8 cm^{-1}.

Powder X-ray diffraction (PXRD): Structural analysis of the prepared PTI-MX was performed with a Bruker D2 Phaser X-Ray powder diffractometer (XRD) in Bragg-Brentano geometry. X-rays were generated by a Cu $K_{\alpha1+2}$ source at 30 kV operating voltage and collected with a LynxEye detector.

Photoluminescence (PL): Edinburgh Instruments FLS 980 spectrometer. Films were dropcasted from ethanol suspensions on quarz substrates PGO 10x10x0.5 mm.

X-ray photoelectron spectroscopy (XPS): JOEL JPS-9030 photoelectron spectrometer with an Al kα (1486 eV) excitation source and a monochromator. Quantitative comparison of nitrogen and carbon in single spectra were performed by signal integration after background subtraction of a Shirley function. The samples were prepared on ITO coated glass substrates to minimize charging.

Scanning electron microscopy and energy-dispersive X-ray spectroscopy: We recorded the SEM images with a GeminiSEM 500 electron microcsope (Carl Zeiss Microscopy GmbH, Germany). The elemental analysis was carried out using an energy-dispersive X-ray spectrometer (EDX, XFlash 6130, Bruker GmbH, Germany) using the same machine. Optical microscopy was performed with an Olympus LEXT laser scanning microscope.

Single-crystal X-ray diffraction (sc-XRD): The data was collected at the beamline MX14.2 of the BESSYII synchrotron and processed using XDS.[25,26] The structure was solved by

intrinsic phasing method (SHELXT-2013)[27] and refined by full matrix least square procedures based on F2 with all measured reflections (SHELXL-2014)[27] in the graphical user interface (OLEX2)[28] with anisotropic temperature factors for all non-hydrogen atoms. If applicable and mentioned, remaining electron density originating from solvent molecules that could not be refined were masked using the solvent mask tool implemented in OLEX2.[29] Deposition Number 2095271 contains the supplementary crystallographic data for this paper. These data are provided free of charge by the joint Cambridge Crystallographic Data Centre and Fachinformationszentrum Karlsruhe Access Structures service.

Scanning force microscopy (SFM): The imaging was performed in amplitude modulation (AM) mode with a Cypher-ES AFM instrument (Asylum research Oxford Instruments Inc.) using photo-thermal excitation of cantilevers. We used qp-fast – long (Nanosensors) cantilever with the nominal fundamental resonance frequency 250 kHz, and spring constant 15 N/m. The cantilever was excited on its third eigenmode at about 5.2 MHz. The setpoint amplitudes were 70 mV. The cantilever was cleaned with air plasma with a Zepto instrument (Diener electronics Inc.) at 50% power for one minute. The sample cell was purged with dry nitrogen (Linde group, 99.999% purity as specified by the manufacturer). Samples were prepared on blue scotch tape (1007R Silicone-Free Blue Adhesive Plastic Film, sps-europe), sonicated in ethanol and glued to a magnetic sample holder with epoxy resin. The images were post processed and plotted in Gwiddyon using the align rows function. The Connolly surface was created in Materials Studio 7.0 with a 001 surface slab of the melem hydrat crystal structure.

Nitrogen (N_2) sorption measurements and BET surface area calculations:

Gas adsorption isotherms were collected using a Micromeritics ASAP 2020 Accelerated Surface Area and Porosity Analyzer. 10-40 mg samples were transferred to dried and tared analysis tubes equipped with filler rods and capped with a seal frit. The samples were heated

to 40 °C at a rate of 1 °C/min and evacuated at 40 °C for 60 min, then heated to 120 °C at a rate of 1 °C/min heat and evacuated at 100°C for 10 h. The tube was weighed again to determine the mass of the activated sample and transferred to the analysis port of the instrument. 99.999% purity N2 was used for all adsorption measurements. N2 isotherms were measured from a relative pressure p/p0 of 0.01 to 1 in a liquid nitrogen (77 K) bath. Brunauer-Emmett-Teller (BET) surface areas were calculated from the N2 isotherm at 77 K within the pressure range p/p0 of 0.05 – 0.07.

$$Q_{monolayer} = \frac{1}{k+d}$$

$$\mathrm{St} = \left(\frac{\mathrm{Q}_{monolayer} \, \frac{\mathrm{cm}^3}{\mathrm{g}}}{\left(22414 \, \frac{\mathrm{cm}^3}{\mathrm{mol}}\right)} \right) * \ \mathrm{NA\ mol}^{-1} * \mathrm{N}_{cs}\ \mathrm{m}^2 \ = \ 4.35 \ * \ \mathrm{Q}_{monolayer} \ \frac{\mathrm{m}^2}{\mathrm{g}}$$

St: Surface area in m^2 g^{-1}

$Q_{monolayer}$: quantity of adsorbed nitrogen in cm^3 g^{-1}

k: slope of linear fit of (p/p°)/(Q(1-(p/p°)) plotted versus (p/p°)

d: y axis intersection of linear fit of (p/p°)/(Q(1-(p/p°)) plotted versus (p/p°)

NA: Avogadro constant 6.022 10^{23} mol^{-1}

N_{cs}: Nitrogen cross section 1.62 10^{-19} m^2

(Peter Klobes, Klaus Meyer Ronald G. Munro, Porosity and specific surface area Measurements for solid materials, 2006, Washington, National Institute of Standards and Technology)

ssNMR: Cross polarization magic-angle spinning (CP-MAS) solid-state NMR spectra were recorded on a Bruker Avance 400 MHz spectrometer operating at 100.6 MHz (^{13}C) and 40.52 MHz (^{15}N).

Low-dose, high-resolution transmission electron microscopy: The sample was dispersed in Methanol (~0.6 mg/mL) with a micropipette and 0.5-1 µL were dropcast in the middle of a

holy carbon TEM grid (Plano S147-2). The TEM grids were loaded into a cryogenic transfer holder (Gatan 914, Gatan, Munich, Germany) at room temperature and transferred to the TEM. Once in the TEM, the holder was cooled down with liquid nitrogen and imaging was performed with a low dose acquisition scheme using SerialEM (doi: 10.1016/j.jsb.2005.07.007) on JEM-2100 (JEOL GmbH, Eching, Germany) operated at 200 kV and equipped with a 4 k × 4 k CMOS digital camera (TVIPS TemCam-F416). HRTEM images were acquired at a magnification of 500,000×, corresponding to a pixel size of 0.23 Å at the specimen level, while keeping the total electron dose below 20 e $Å^{-2}$. All imaging was carried out at temperatures around 90 K.

Hydrogen evolution reaction: 20 mg 500 °C, 12 h iodide eutectic condensation product is added to a 10 vol% 15 ml solution of the sacrificial agent triethanolamine in water. 0.2 ml of a 1 mg/mL H_2PtCl_6 solution is added as co-catalyst and the reaction mixture is argon purged for 10 min while stirring. The reaction mixture is illuminated with a 400 W xenon lamp without cutoff filter and samples of the headspace are extracted through a septum at hourly intervals for five hours. The hydrogen content of the collected samples was determined by gas chromatography. Hydrogen content is under the limit of detection. Please find details about the setup here: doi: 10.33774/chemrxiv-2021-q1b4b.

Table S1. Combustion elemental analysis of of products obtained from the LiI/KI eutectic at various reaction conditions compared to a PTI/Br sample from a LiBr/KBr eutectic.

sample	N wt%	C wt%	H wt%
LiBr/KBr, 550 °C, 48 h	43.118	25.958	1.059
LiI/KI, 550 °C, 48 h	38.100	40.009	2.688
LiI/KI, 550 °C, 12 h	53.294	38.840	2.452
LiI/KI, 500 °C, 24 h	57.393	30.387	3.384
LiI/KI, 450 °C, 12 h	58.287	30.163	3.432

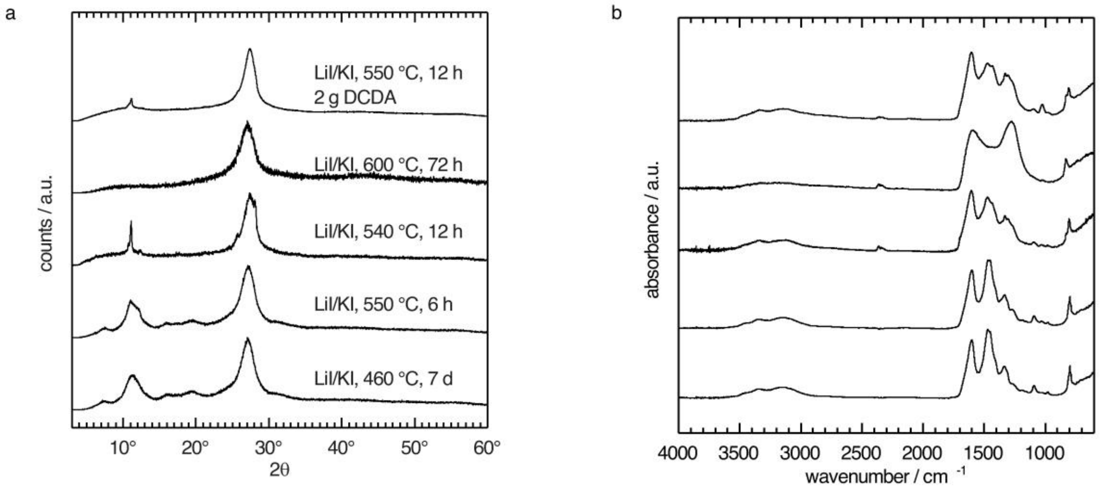

Figure S1. PXRD and FT-IR spectra of additional reaction conditions.

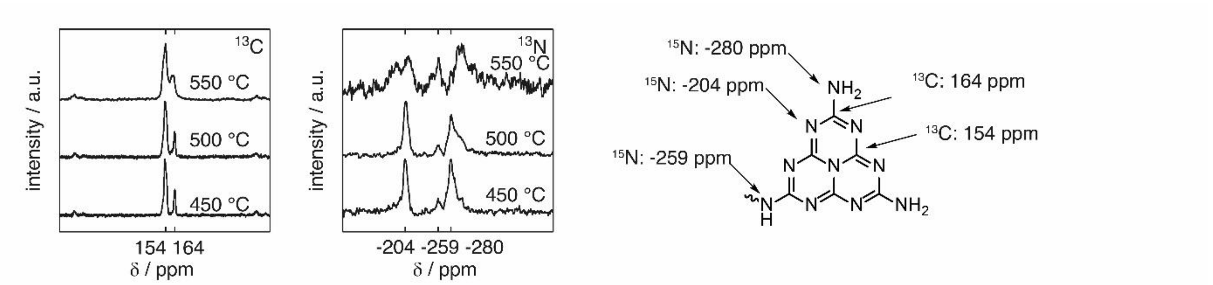

Figure S2. ^{13}C and ^{15}N Solid state NMR of carbon nitride condensation products from 550 °C, 12 h (top line), 500 °C 24 h (middle line) and 450 °C 12 h (bottom line). The bands are assigned to a melem unit in a structural model on the right side of the spectra.

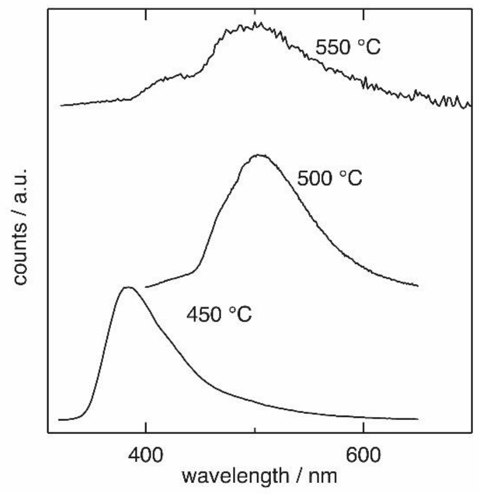

Figure S3. Photoluminescence spectra of CN condensation products from iodide eutectic. Product formed at 450 °C excited at 310 nm closely resembles the emission of melem with a emission maximum at 380 nm (bottom line). The emission of product formed at 500 °C excited at 370 nm is red shifted with an emission maximum at 500 nm (middle line). The product formed at 550 °C excited at 310 nm follows the trend of the broadening emission at 500 nm.

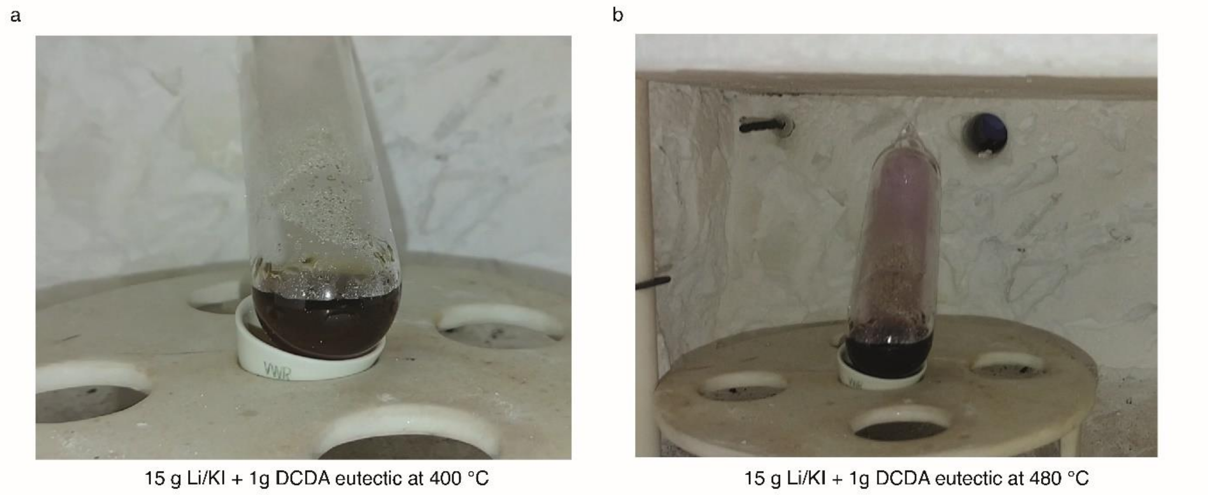

Figure S4. Images of reaction mixtures in a furnace at 400 and 480 °C.

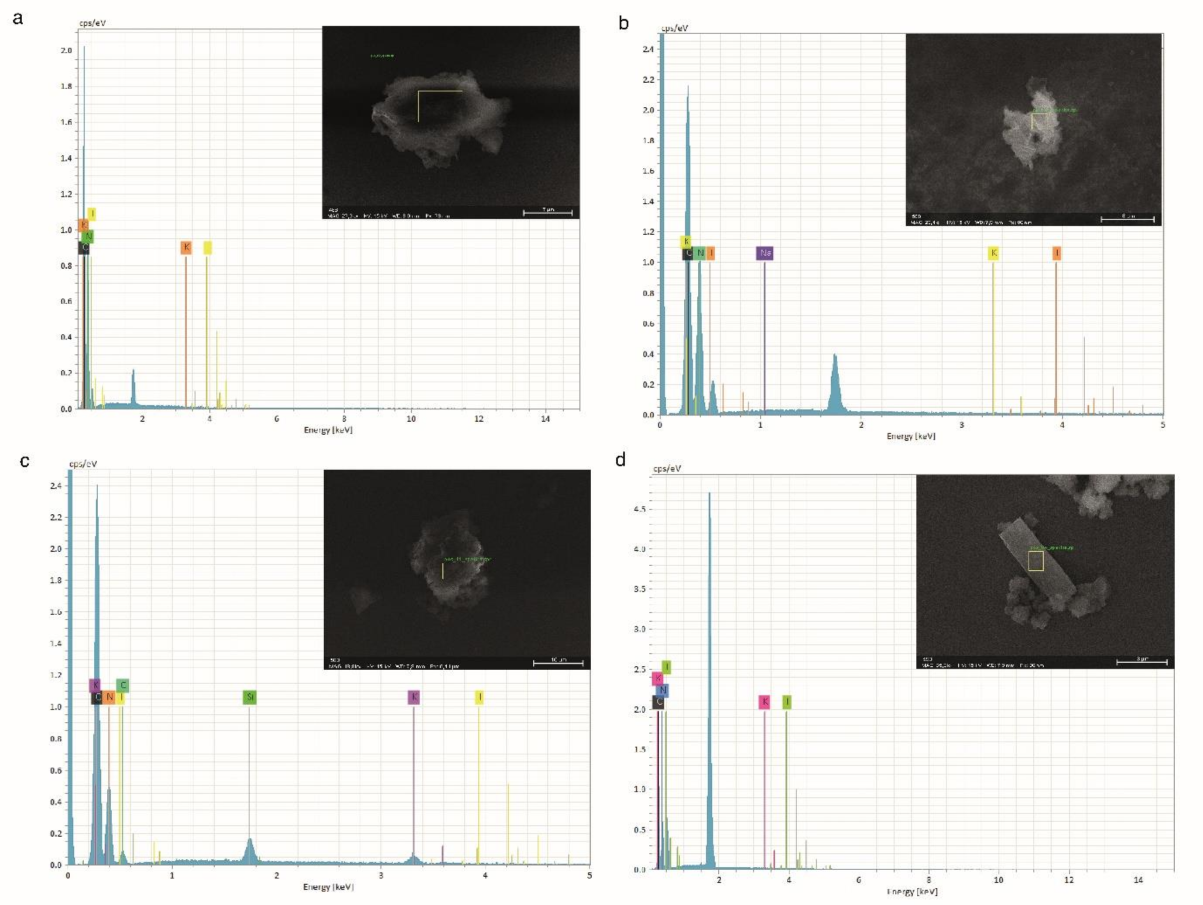

	nitrogen at%	carbon at%	potassium at%	iodine at%
a) 450 °C, 12 h	63±9	37±4	0±0	0±0
b) 500 °C, 12 h	62±8	38±4	0±0	0±0
c) 550 °C, 12 h, amorph	49±7	50±5	0.3±0.1	0±0
d) 550 °C, 12 h, crystal	65±10	35±4	0±0	0±0

Figure S5. Energy dispersive X-ray spectroscopy of condensation products. Atomic percentages of nitrogen and carbon reflect the combustion analysis well. The increased carbon content of the 550 °C, 12 h can be attributed to the brownish amorphous phase.

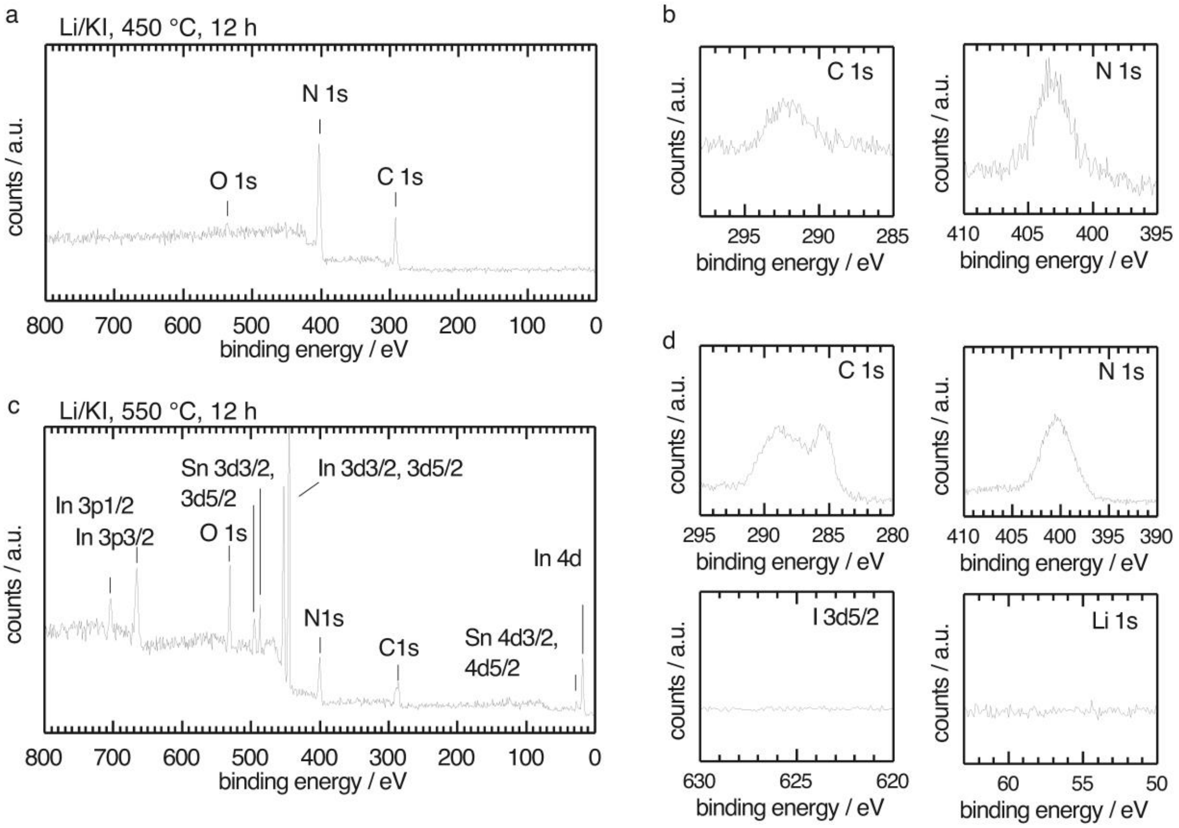

Figure S6. XPS spectra of condensation product from 450 °C and 550 °C. The concentration of the salt melt ions in the sample surface of processed films on indium-tin oxide after workup is under the detection limit. a) XPS survey of CN condensation product from 450 °C. Only carbon nitrogen and oxygen are present. b) XPS spectra of C 1s and N1s. The atomic percentages of carbon and nitrogen are in well accordance with the values found by combustion analysis and EDX nitrogen: 65 at% carbon 35 at%. Due to a film thickness of around 1-10 µm no indium and tin signals are obtained. However the absolute shift is most likely influenced by surface charging (compare e.g. the N 1s spectra). c) The XPS spectrum of the product obtained from 550 °C has as well no ion contributions from the salt melt (LiI/KI). Due to reduced film thickness, in this sample, charging was successful reduced. However, full surface coverage was not achieved and hence contributions of elements of the indium tin oxide substrate are captured in the survey spectrum. d) The C 1s spectrum shows similar trends as observed for carbonization in poly(triazine imide) (doi: 10.1002/anie.202111749). A strong signal at 285 eV arises that can be interpreted as CC environments. The carbon rich property is also reflected by comparison of the atomic percentages of nitrogen: 47 at% and carbon 53 at% to the condensation at lower temperature. Again these values are in good accordance with combustion analysis and EDX.

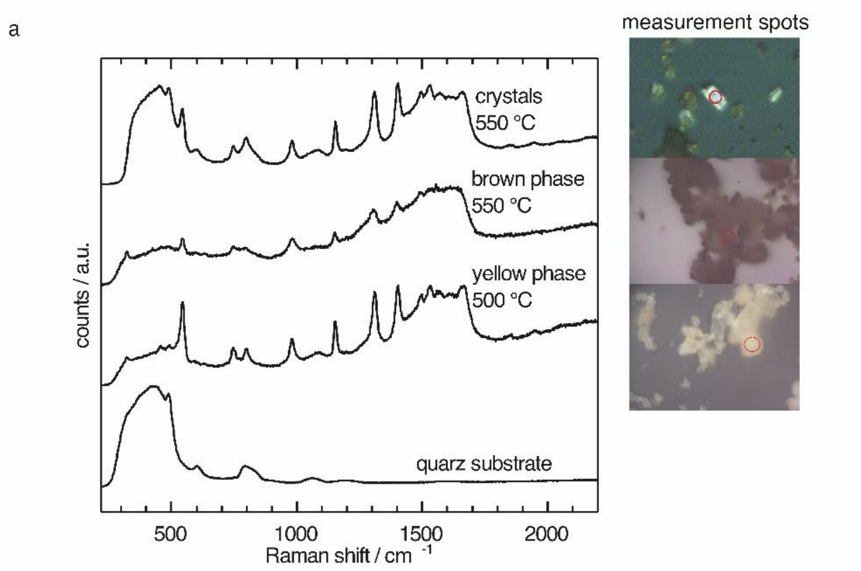

b

ν (observed this work)	ν reference	assignment
1661 (w)	1677 (w)	E′
1571 (w)	1581 (w)	E′
1530 (w)		
1494 (w)		
1401 (s)		
1310 (s)		
1197 (vw)		
1155 (s)	1155 (s)	A1′
980 (s)		
750 (w)		
545 (s)	545 (s)	A1′

Figure S7. UV-Raman spectrum with 255 nm excitation wavelength of DCDA condensation products in LiI/KI eutectics at 500 °C and 550 °C. a) rectangular crystals from 550 °C, 12 h reactions show well resolved Raman bands on a quarz substrate (first line). The band positions of the coevolved brown phase measured on a cleaved mica substrate are closely related to the bands observed in the crystalline phase. Product from 500 °C 12 reaction shows the same dominant Raman bands as the product phases obtained from 550 °C. The observed Raman bands are compared to literature values for melem. (doi: 10.1021/ja0357689). The high similarity of the spectra of the melem crystals obtained from the 550 °C sample and the yellow phase from 500 °C further strengthens the hypothesis that the observed changes in the PXRD are due to phase changes of melem crystals.

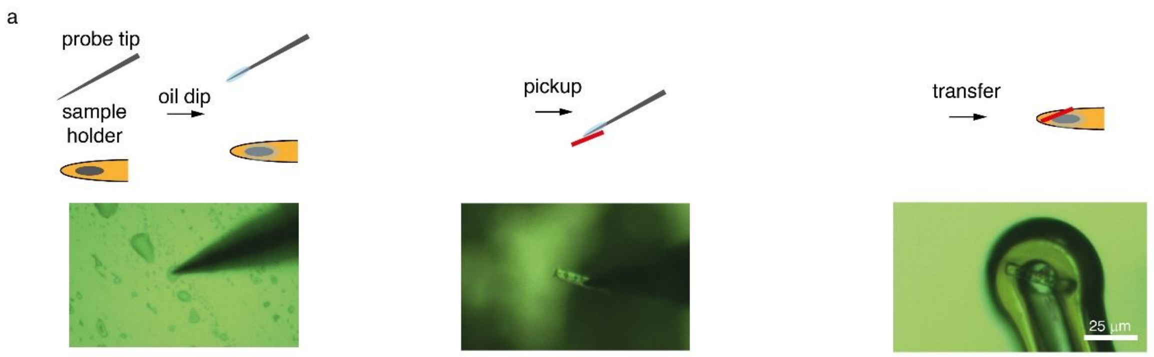

Figure S8. Single crystal X-ray sample preparation. a) pick up transfer of microcrystals in presence of abundant amorphous phase. To be able to mount the crystals even though their small size and the presence of the second brown phase, a pickup transfer with micro-actuators is applied (IMINA technologies). A micro actuator tip is dipped into an oil-drop on a microscopy slide, resulting in better adhesion properties of the tip. The tip with the oil film is used to pick up the micro crystallites. A sample holder with a 10 µm loop is also dip-coated with oil. Then the crystallites were carefully transferred to the sample holder.

Table S2. Crystallographic data for melem hydrate ($C_6N_7(NH_2)_3{\cdot}H_2O$) obtained from the ionothermal condensation of dicyandiamide in LiI/KI eutectic salt melt at 550 °C, 12 h.

Identification code	melem hydrate
Empirical formula	$C_6H_8N_{10}O$
Formula weight	236.22
Temperature/K	100 K
Crystal system	monoclinic
Space group	$P2_1/c$
a/Å	8.6040(17)
b/Å	16.630(3)
c/Å	6.8840(14)
α/°	90
β/°	111.91(3)
γ/°	90
Volume/$Å^3$	913.8(4)
Z	4
$\rho_{calc}g/cm^3$	1.717
μ/mm^{-1}	0.132
F(000)	488.0
Crystal size/mm^3	0.1 × 0.04 × 0.01
Radiation	synchrotron (λ = 0.799897 Å)
2Θ range for data collection/°	4.898 to 59.504
Index ranges	$-11 \leq h \leq 11$, $-22 \leq k \leq 22$, $-8 \leq l \leq 8$
Reflections collected	15426
Independent reflections	2333 [R_{int} = 0.1298, R_{sigma} = 0.0782]
Data/restraints/parameters	2333/6/175
Goodness-of-fit on F^2	1.024
Final R indexes [I>=2σ (I)]	R_1 = 0.0797, wR_2 = 0.2012
Final R indexes [all data]	R_1 = 0.1656, wR_2 = 0.2574
Largest diff. peak/hole / e $Å^{-3}$	0.39/-0.37

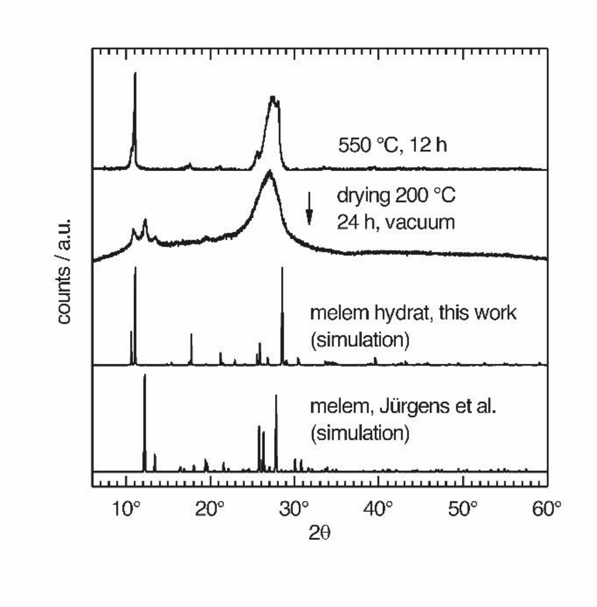

Figure S9. PXRD of condensation product from 550 °C, 12 h (first line) compared to PXRD after vacuum drying (second line). The experimentally obtained diffractograms are compared with PXRD simulations of the structural data files of melem hydrat (this work, third line) and the structure of solvent free melem as obtained by Jürgens et al. (fourth line).

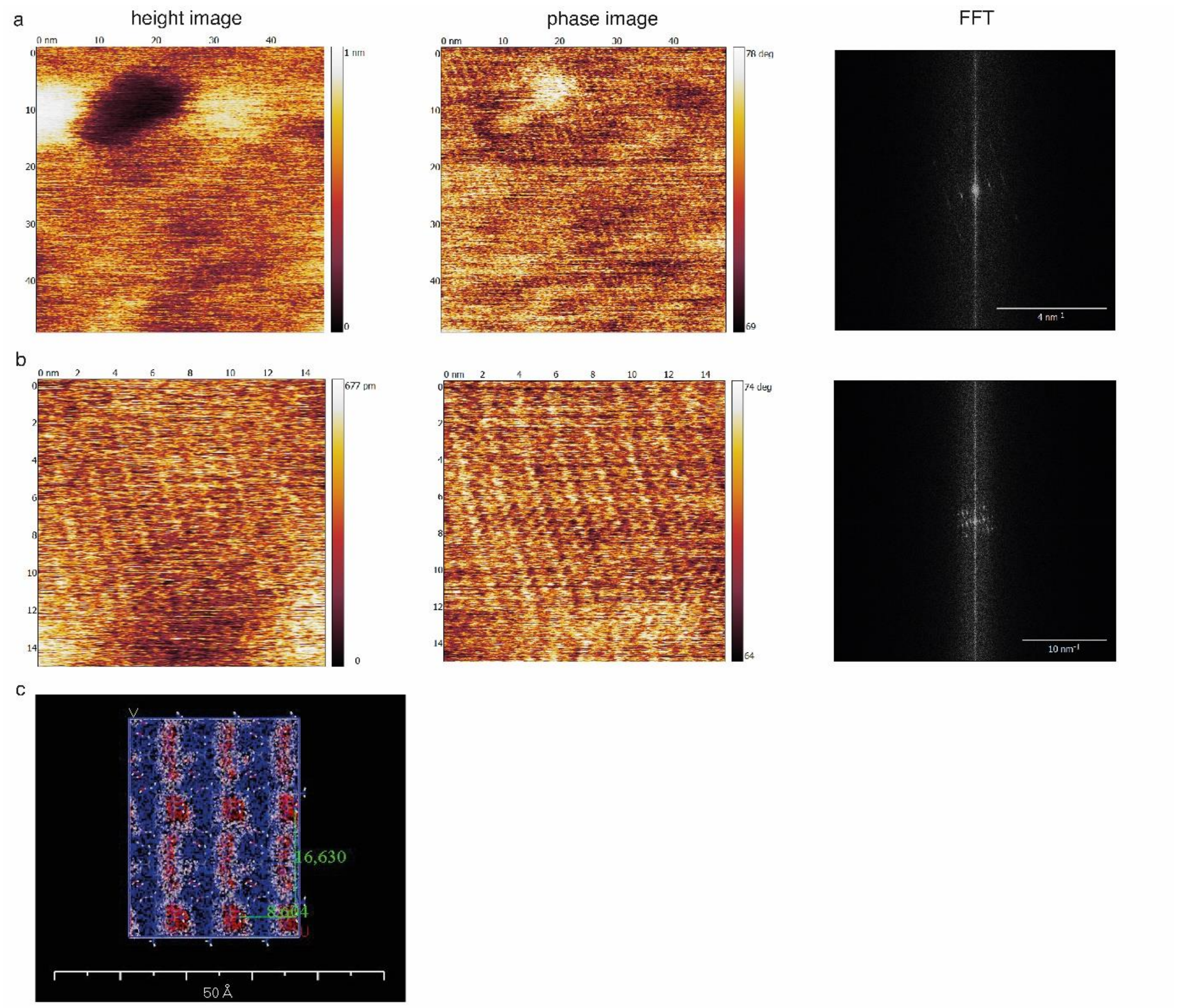

Figure S10. SFM images obtained from melem hydrat crystallite surfaces. Predominantly occurring periodic distances are 0.7 nm and 1.7 nm. a) height and phase image of melem hydrat crystal surface. Fast Fourier transform analysis of the phase images exhibit spots at the spatial frequencies in relation to the image center. b) after decreasing the scan size the periodic distances are still present. c) 001 Connolly surface of melem hydrat crystal structure. The dark-red marked areas contain amine residues pointing out of the surface which are in direct contact with the spheric “connolly-probe”. The distances and angles of the Connolly surface closely resemble the observed patterns. The small deviations can be due thermal drift of the sample and surface adsorbents.

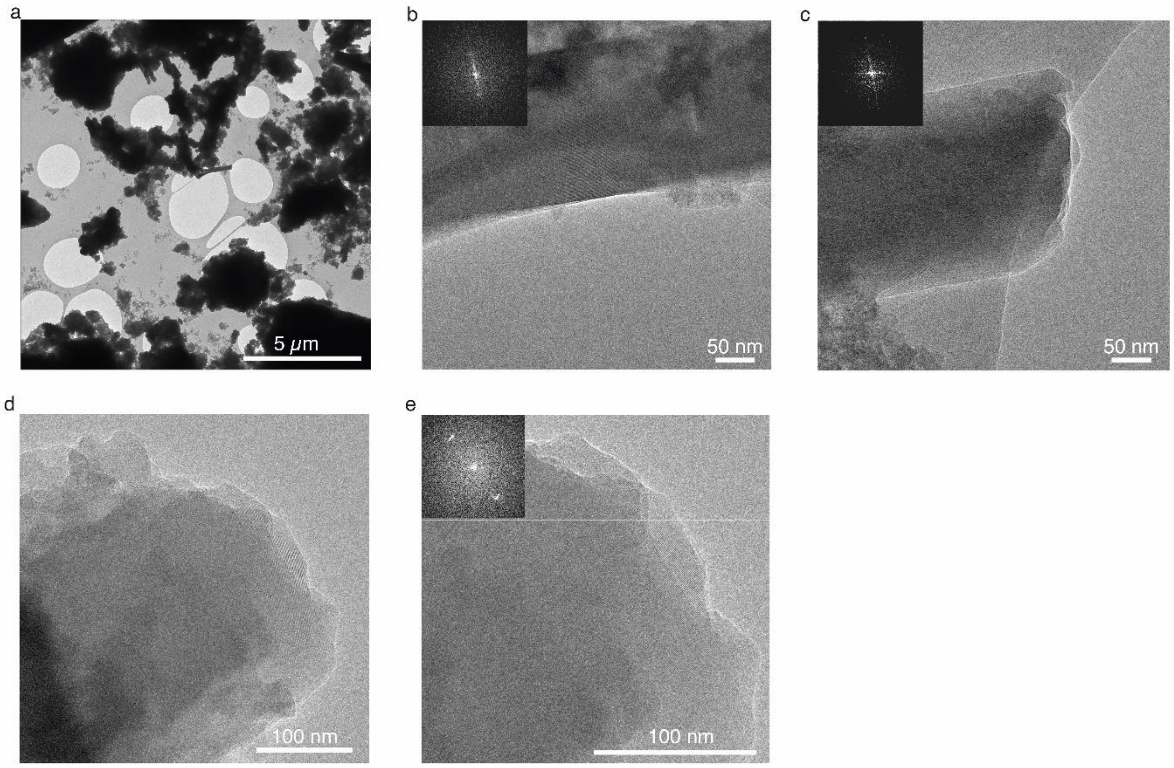

Figure S11. Cryo HR-TEM (200 kV acceleration voltage) images and fast Fourier transform of an elongated rectangular carbon nitride crystal exhibiting lattice fringes with a periodicity of 0.8 nm. This distance is interpreted as the 100 d-spacing of melem hydrat.

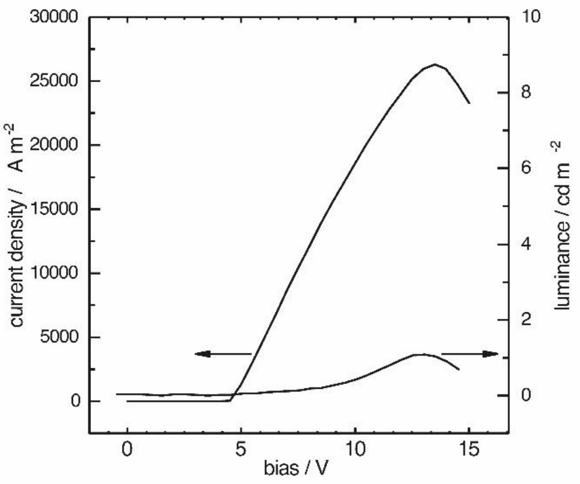

Figure S12. IV characteristics and luminance of OLED constructed from 500 °C condensation product. The device shows a steep diode like onset at 4.5 V in forward bias direction. At 13 V one observes a maximum luminance of 1 cd m^{-2}. Electroluminescence spectra were not obtained since the luminescence was not high enough for the set up that has been employed.

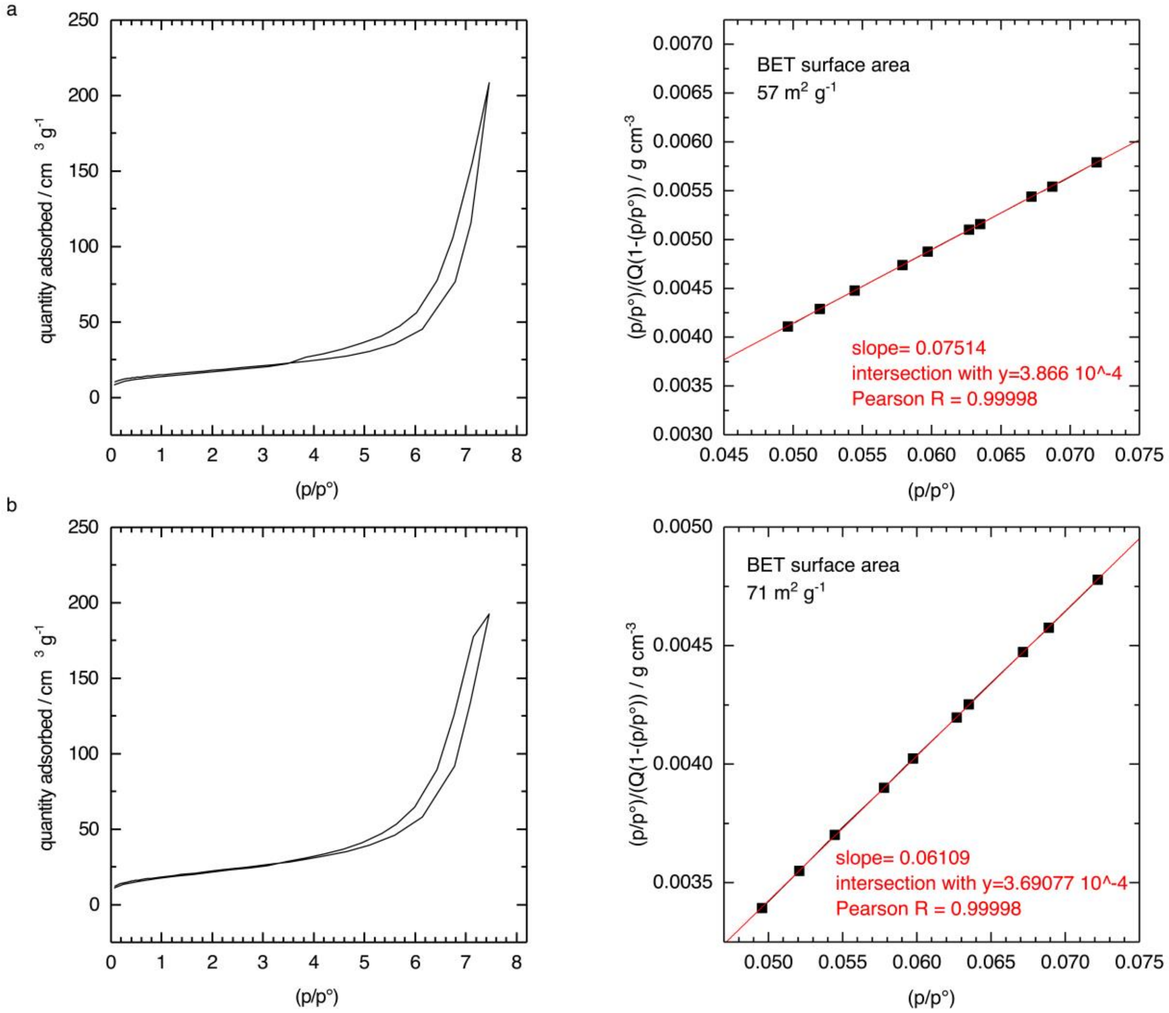

Figure S13. Nitrogen de- and adsorption isotherms from a) 450 °C, 12h and b) 550 °C, 12h CN condensation products from a LiI/KI eutectic salt melt.
